# COVID‐19‐related posttraumatic stress disorder in adults with lived experience of psychiatric disorder

**DOI:** 10.1002/da.23262

**Published:** 2022-05-10

**Authors:** Catrin Lewis, Katie Lewis, Alice Roberts, Claudia Evison, Bethan Edwards, Ann John, Keith Lloyd, Holly Pearce, Rob Poole, Natalie Richards, Catherine Robinson, Ian Jones, Jonathan I. Bisson

**Affiliations:** ^1^ Division of Psychological Medicine and Clinical Neurosciences, National Centre for Mental Health Cardiff University School of Medicine Cardiff UK; ^2^ National Centre for Mental Health, Population Data Science Swansea University Medical School Swansea UK; ^3^ National Centre for Mental Health, Centre for Mental Health and Society Bangor University Bangor UK; ^4^ Social Care and Society, School of Health Sciences University of Manchester Manchester UK

**Keywords:** collective trauma, COVID‐19, ICD‐11, PTSD

## Abstract

**Background:**

Prevalence estimates of COVID‐19‐related posttraumatic stress disorder (PTSD) have ranged from 1% to over 60% in the general population. Individuals with lived experience of a psychiatric disorder may be particularly vulnerable to COVID‐19‐related PTSD but this has received inadequate attention.

**Methods:**

Participants were 1571 adults with lived experience of psychiatric disorder who took part in a longitudinal study of mental health during the COVID‐19 pandemic. PTSD was assessed by the International Trauma Questionnaire (ITQ) anchored to the participant's most troubling COVID‐19‐related experiencevent. Factors hypothesised to be associated with traumatic stress symptoms were investigated by linear regression.

**Results:**

40.10% of participants perceived some aspect of the pandemic as traumatic. 5.28% reported an ICD‐11 PTSD qualifying COVID‐19 related traumatic exposure and 0.83% met criteria for probable ICD‐11 COVID‐19‐related PTSD. Traumatic stress symptoms were associated with younger age, lower income, lower social support, and financial worries, and lived experience of PTSD/complex PTSD. Depression and anxiety measured in June 2020 predicted traumatic stress symptoms at follow‐up approximately 20 weeks later in November 2020.

**Conclusions:**

We did not find evidence of widespread COVID‐19‐related PTSD among individuals with lived experience of a psychiatric disorder. There is a need for future research to derive valid prevalence estimates of COVID‐19‐related PTSD.

## 1 INTRODUCTION

The COVID‐19 pandemic has been associated with a range of psychological sequelae, including posttraumatic stress disorder (PTSD) (Vindegaard & Benros, 2020). The estimated prevalence of COVID‐19‐related PTSD within study samples has ranged from 1% to 67% in the general population of some countries (Yuan et al., 2021; Zhang et al., 2021). A high proportion of these studies reported traumatic stress symptoms rather than applying the full diagnostic criteria and most made general point‐prevalence estimates of PTSD during the pandemic, rather than anchoring measurement to COVID‐19‐related trauma. A review that conducted subgroup analyses by region, measurement scale, and population (e.g., the elderly), failed to reduce the heterogeneity based on these factors and provide an explanation for the wide‐ranging prevalence estimates (Zhang et al., 2021). Among healthcare workers, reviews of the literature have reported prevalence estimates for traumatic stress symptoms that ranged between 2% and 73%, with the highest estimates from studies in China during the initiation and acceleration phase of the outbreak (Al Falasi et al., 2021; d'Ettorre et al., 2021).

Despite these findings, the validity of PTSD as a construct in the context of COVID‐19 has been a subject of debate. The pandemic creates a risk of exposure to PTSD qualifying events, such as experiencing the life‐threatening symptoms of COVID‐19, or of witnessing others being critically unwell or dying (Bridgland et al., 2021). These experiences may be considered PTSD qualifying according to both DSM‐5 (“exposure to actual or threatened death, serious injury or sexual violence”) (APA, 2013) and ICD‐11 (“exposure to an extremely threatening or horrific event or series of events”) definitions of qualifying events (WHO, 2018). It has been argued that living through the pandemic may precipitate PTSD in itself, at least according to ICD‐11 criteria (Shevlin et al., 2020). However, this is a contentious issue and there is no agreement that the pandemic is an eligible traumatic stressor (Norrholm et al., 2021).

To some degree, variability in COVID‐19‐related PTSD prevalence estimates reflect true differences across countries, populations, and settings. Some variability would also be expected due to differences in diagnostic criteria and measurement tools. However, the extent of the variability is driven, at least in part, by methodological limitations. Most studies have failed to anchor PTSD symptoms to the pandemic, or they have anchored responses to the pandemic as a single entity, without determining whether a PTSD qualifying event was experienced (Asmundson & Taylor, 2021). At the upper end of the range of reported estimates, the prevalence of COVID‐19‐related PTSD is higher than reported within study samples exposed to terror attacks, mass shootings, and natural disasters (Santiago et al., 2013). It can be argued that these prevalence rates are artefacts of the methods utilised to derive the estimates, rather than a true reflection of the scale of the issue. There is a need to establish more valid estimates by determining whether COVID‐19‐related traumatic stressors are PTSD qualifying and assessing symptoms anchored to these events over a relevant timeframe.

Despite the potentially heightened risk of negative outcomes from the COVID‐19 pandemic for those with existing psychiatric disorders, we know far less about its impact than in the general population (Brown et al., 2020). Accordingly, our aim was to investigate COVID‐19 related PTSD within a sample of participants with lived experience of a psychiatric disorder, to (1) determine whether self‐reported COVID‐19 related events were PTSD qualifying according to ICD‐11 and/or DSM‐5 criteria; (2) using an ICD‐11 self‐report measure of PTSD (the International Trauma Questionnaire [ITQ; Cloitre et al., 2018]), to estimate the prevalence of probable COVID‐19‐related PTSD anchored to these events; and (3) determine key factors associated with COVID‐19‐related traumatic stress symptoms.

## 2 MATERIALS AND METHODS

### 2.1 Data source

Data were obtained from a longitudinal study of mental health during the COVID‐19 pandemic conducted by the National Centre for Mental Health (NCMH). NCMH is a Welsh Government‐funded Research Centre that operates in partnership with the National Health Service (NHS) across Wales and England. NCMH hosts a cohort of participants with lived experience of a psychiatric disorder who were recruited on a rolling basis from 2011 using a variety of systematic approaches in primary and secondary health care services, as well as via advertisements and through third‐sector organisations. Due to the sampling strategy, it is not a nationally representative population‐based cohort, but a targeted recruitment of participants with psychiatric disorder. It is composed of participants with lived experience including but not limited to, neurodevelopmental disorders (e.g., attention‐deficit hyperactivity disorder [ADHD]), depression, anxiety, schizophrenia, bipolar disorder, eating disorders, personality disorder, and PTSD. Informed consent was obtained from all participants and study procedures were given a favorable ethical opinion by Wales Research Ethics Committee 2.

### 2.2 Sample

In June 2020, all members of the NCMH cohort aged 18 years or older with an email address and lived to experience of a psychiatric disorder who consented to be contacted for future research (*n* = 10,017/20,117) were invited to join the online COVID‐19 study. This baseline COVID‐19 survey included questions on demographic variables, mental health, and the COVID‐19 pandemic. In November 2020, a follow‐up survey was sent to the 3712 participants who completed the baseline survey, which repeated questions from the initial survey and asked additional questions specifically related to traumatic stress. The sample for analysis were 1571 participants who completed the section on COVID‐19‐related trauma in the follow‐up survey.

### 2.3 Demographic and clinical information

Self‐reported age, gender, income, employment, and key worker status were captured in the baseline survey. The term “key worker” was widely used in the United Kingdom during the COVID‐19 pandemic to indicate roles that were vital to society (e.g., in health and public services), which were exempt from guidance to work from home. Financial changes during the pandemic were determined in the follow‐up survey, in addition to establishing COVID‐19‐related worry about finances on a 5‐point scale ranging from “not at all worried” to “very worried.” Mental health diagnoses were collected with the question “what mental health or neurodevelopmental diagnosis or condition have you been given or received treatment for?” with a list of options (see Supporting Information Material). Diagnoses were grouped into anxiety, depression, obsessive compulsive disorder (OCD), bipolar disorder, schizophrenia/psychosis, PTSD, complex PTSD (CPTSD), eating disorder, personality disorder, alcohol/other drug misuse, autism spectrum disorders, and ADHD.

### 2.4 COVID‐19‐related information

Participants reported symptoms of COVID‐19 and if they tested positive (yes/no). They also reported medical conditions that put them at risk of severe infection from COVID‐19 from a list of options such as diabetes or heart disease (see Supporting Information Material). Participants were asked how socially supported they felt in the past 2 weeks on a 5‐point scale ranging from “very poorly” to “very well.”

### 2.5 Trauma exposure and PTSD

Participants were asked if they found any aspect of the COVID‐19 pandemic traumatic (yes/no). If “yes,” they gave a free‐text description of the most troubling experience. Traumatic experiences were coded by two researchers (CL and AR) against a list compiled for the study (see Table 1). The same two researchers independently judged whether each free‐text description met the traumatic stressor criterion of (1) ICD‐11 (“exposure to an extremely threatening or horrific event or series of events”) (WHO, 2018) and (2) DSM‐5 (“exposure to actual or threatened death, serious injury or sexual violence”) (APA, 2013). If coders disagreed, this was discussed with a third researcher (JB) and a consensus was reached. All participants who endorsed a traumatic experience responded to the PTSD subscale of the ITQ (Cloitre et al., 2018) anchored to the traumatic exposure. They indicated how much they had been bothered by six core PTSD symptoms in the past month using a 5‐point Likert scale ranging from 0 (“not at all”) to 4 (“extremely”). Additional items capture functional impairment. Participants were considered to fulfil the criteria for probable COVID‐19 related ICD‐11 PTSD if a qualifying trauma was reported, a score of ≥2 (“moderately”) was obtained for at least one of two symptoms from each of the three symptom clusters, and at least one of the functional impairment items was endorsed.

**Table 1:** Sample characteristics.

Variable	*N* (%) or mean (SD)
Age	47.12 (15.43)
Gender	
Female	1191 (75.81%)
Transgender female	3 (0.19%)
Male	340 (21.64%)
Transgender male	9 (0.57%)
Gender variant/nonconforming/nonbinary	22 (1.4%)
Missing	6 (0.38%)
Ethnicity	
White Caucasian	1499 (95.41%)
Ethnic minority	53 (3.39%)
Missing	19 (1.2%)
Employment	
Employed	825 (52.51%)
Retired	269 (17.12%)
Student	120 (7.64%)
Unemployed	355 (22.6%)
Missing	2 (0.13%)
Household income	
Up to £10,000	362 (23.05%)
£10,000–£20,000	253 (16.10%)
£20,000–£30,000	241 (15.34%)
Over £30,000	532 (33.86%)
Missing	183 (11.64%)
Highest level of qualification	
None/less than equivalent to GCSE	36 (2.29%)
GCSE or equivalent	281 (17.89%)
A level or equivalent	302 (19.22%)
Degree or above	835 (53.15%)
Missing	117 (7.45%)
Key worker	
No	1122 (71.42%)
Yes	417 (26.54%)
Missing	32 (2.04%)
Infection from COVID‐19	
Symptoms of COVID‐19	263 (16.76%)
Tested for COVID‐19	172 (10.95%)
Tested positive for COVID‐19	42 (2.67%)
Mental health diagnoses	
Bipolar disorder	215 (13.66%)
Schizophrenia	147 (9.36%)
Depressive disorder	1202 (76.51%)
Anxiety	1366 (86.95%)
Eating disorder	225 (14.32%)
Personality disorder	210 (13.37%)
PTSD	262 (16.68%)
Complex PTSD	89 (5.67%)
Autism spectrum disorders	109 (6.94%)
Worst COVID‐19 traumatic event	
Infection/suspected infection from COVID‐19	14 (2.22%)
Infection/suspected infection of loved one from COVID‐19	41 (6.51%)
Death of a loved one from COVID	13 (2.06%)
Death of a loved one (not from COVID‐19)	21 (3.33%)
Being in a high‐risk group for severe infection	12 (1.90%)
Working in a role with exposure to the virus	27 (4.29%)
Generalised worry about the pandemic	192 (30.48%)
Lockdown and social distancing restrictions	133 (21.11%)
Worry about finances/employment	17 (2.70%)
Government response to the pandemic	8 (1.27%)
Behaviour of others during the pandemic	19 (3.02%)
Use of face coverings	28 (4.44%)
Exposure to news or media coverage	27 (4.29%)
Changes in access to medical care	24 (3.81%)
Other	25 (3.97%)
Missing	29 (4.60%)

### 2.6 Symptoms of current depression

Symptoms of current depression were measured by the 9‐item Patient Health Questionnaire‐9 (PHQ‐9; Kroenke & Spitzer, 2002). Respondents indicated the frequency of symptoms in the previous 2 weeks on a 4‐point Likert scale, ranging from 0 (not at all) to 3 (nearly every day). A cut off score of 10 or more is taken to indicate a possible episode of depression (Manea et al., 2012) (Table 2).

**Table 2:** Results of regression analyses.

Variable	*B*	95% CI	*p*	*B*	95% CI	*p*
Age	−0.05	−0.09 to −0.02	.002	−0.06	−0.10 to −0.02	.004^a^
Gender	0.66	−0.66 to 1.97	.326	0.78	−0.56 to 2.12	.253
Low income	2.86	1.80–3.92	.000	3.03	1.93 to 4.13	.000^a^
Social support	−2.05	−3.05 to −1.04	.000	−2.42	−3.50 to −1.35	.000^a^
Keyworker status	−1.18	−2.35 to −0.01	.049	−1.13	−2.41 to 0.16	.085
Financial worry	3.41	2.35–4.47	.000	2.98	1.82–4.13	.000^a^
Risk of severe infection	1.54	0.45–2.62	.006	1.19	0.01–2.37	.048
Lived experience PTSD/CPTSD	3.30	−0.13 to 2.58	.077	0.97	−0.50 to 2.44	.000
PHQ‐9	0.41	0.35–0.47	.000	0.36	0.29–0.43	.000^a^
GAD‐7	0.62	0.54–0.68	.000	0.58	0.50 –0.66	.000^a^

*Note*: Age—continuous; gender coded as 0 = male, 1 = female; income coded as 1 = gross family income under £20,000 a year, 0 = household income £20,000 or more; social support coded as 1 = socially supported by friends well/very well, 0 = very poorly/poorly/neither poorly nor well; keyworker status coded as 1 = yes, 0 = no; financial worry—1 = worried/very worried, 0 = not at all worried/a little worried/somewhat worried; risk of severe infection (endorsed a relevant physical health condition) coded as 1 = yes, 0 = no; lived experience of PTSD/CPTSDcoded as 1 = yes, 0 = no.

Abbreviations: GAD‐7, Generalised Anxiety Disorder 7; PHQ‐9, Patient Health Questionnaire 9.

^a^ Variables that survived adjustment for multiple testing.

### 2.7 Symptoms of current anxiety

Symptoms of current anxiety were measured by the 7‐item Generalised Anxiety Disorder Assessment‐7 (GAD‐7; Spitzer et al., 2006). Respondents indicated the frequency of anxiety symptoms in the previous 2 weeks on a 4‐point Likert‐scale, ranging from 0 (not at all) to 3 (nearly every day). A cut off score of 10 or more is taken to indicate a possible diagnosis of generalised anxiety disorder (GAD) (Spitzer et al., 2006).

### 2.8 Statistical procedures

All analyses were conducted using Stata version 16 (StataCorp, 2019; Stata Statistical Software: Release 16; StataCorp LLC.). Sample characteristics were examined using descriptive statistics. The association between key factors and traumatic stress symptoms were investigated by linear regression with total score on the ITQ as the dependent variable. Based on previous literature, factors hypothesised to be associated with COVID‐19‐related traumatic stress symptoms were: age; gender; income; worry about finances; perceived social support; key worker status; symptoms of anxiety and depression at baseline; and lived experience of PTSD/complex PTSD (CPTSD). Analyses were adjusted for potential confounders (age, gender, and income). The Holm method was used to adjust *p* values to account for multiple testing (Holm, 1979).

## 3 RESULTS

Of the 10,017 participants invited to take part in the baseline survey, 3137 (31%) took part (Lewis et al., 2022). The majority of participants (70.1%) completed the survey in the week commencing June 15th, 2020, with the remainder of the sample completing the survey between June 26th, 2020 and July 30th, 2020. At the time the survey was sent out, restrictions in Wales and England were beginning to ease after 3 months in lockdown. Strict restrictions on indoor mixing remained in place, with bars, restaurants, and entertainment venues still closed. Nonessential retail stores in England reopened on the day the baseline survey was sent out but remained closed in Wales until the following week. The second wave of the survey was sent in November 2020. Most participants (69.9%) completed it between November 5th and November 11th, 2020, with the rest of the sample completing the survey between November 6th, 2020 and January 2nd, 2021. At the time the survey was sent, England and Wales were in lockdown and people were only permitted to leave home for a limited number of reasons, including work (if unable to work from home), education, exercise, and shopping for food and essentials. The sample for analysis were 1571 participants (42.32% of the 3712 sent the survey link) who completed the section on COVID‐19‐related trauma in the follow‐up survey.

### 3.1 Sample characteristics

The mean age of participants was 47.1 (SD = 15.43) and 76.0% (*n* = 1194) were female. Most participants were white Caucasian (95.4%); 52.5% were employed (26.54% as key workers); and all had lived experience of at least one psychiatric disorder. Sample characteristics are presented in Table 1. Using variables collected at the point of entry into the NCMH cohort with complete or near‐complete data, we found nonresponse to the trauma survey was associated with younger age, male gender, never having been employed, minority ethnicity, diagnosis of bipolar disorder, and diagnosis of schizophrenia. We did not find evidence of an association between diagnosis of PTSD and/or CPTSD and nonresponse (see Supporting Information).

### 3.2 COVID‐19‐related trauma exposure

40.10% (*n* = 630) of participants found some aspect of the pandemic traumatic, with generalised worry about the pandemic (12.22%; *n* = 192) and lockdown/social distancing restrictions (8.47%; *n* = 133) being the 'most troubling' experiences reported most frequetly. 5.28% (*n* = 83) of participants reported a traumatic exposure that was qualifying according to ICD‐11 and 3.06% (*n* = 48) according to DSM‐5. Traumas that met criteria according to both ICD‐11 and DSM‐5 included severe symptoms of COVID‐19 and witnessing another person being critically unwell or dying. The different rates of qualifying events according to the two diagnostic systems was largely underpinned by the fact that learning of a loved one dying is required to be violent or accidental to meet Criterion A of DSM‐5.

### 3.3 COVID‐19‐related traumatic stress symptoms and PTSD

One hundred and seventy‐one (10.88%) participants met symptom criteria for PTSD on the ITQ. Only 0.83% (*n* = 13) of these participants reported a traumatic stressor that was judged to meet ICD‐11 criteria for a PTSD qualifying event and therefore screened positive for PTSD.

### 3.4 Depression and anxiety

Within the sample (*n* = 1571) mean (SD) scores on the PHQ‐9 were 12.45 (7.38) in the baseline survey with 56.78% scoring above the clinical cut‐off (≥10) and 12.76 (7.64) at follow up (59.26% above the cut off). Mean (SD) scores on the GAD‐7 were 9.70 (6.15) in the baseline survey with 43.92% scoring above the clinical cut‐off (≥10) and 10.25 (6.33) at follow up (49.40% scoring above the cut‐off). Within the sample of participants who endorsed the pandemic being traumatic (*n* = 630) mean (SD) scores on the PHQ‐9 were 13.75 (7.27) in the baseline survey with 68.38% scoring above the clinical cut‐off and 14.23 (7.40) at follow up (69.30% above the cut off). Mean (SD) scores on the GAD‐7 were 11.21 (6.04) in the baseline survey with 56.18% scoring above the clinical cut‐off (≥10) and 11.94 (6.07) at follow up (62.95% scoring above the cut‐off). Measurement of anxiety and depressive symptoms were not specifically anchored to the pandemic.

### 3.5 Associations between ITQ scores and key factors

After adjustment for potential confounders and multiple testing, there was evidence that higher ITQ scores were associated with younger age, lower income, lower social support, financial worries, and lived experience of PTSD/CPTSD. Higher levels of depression and anxiety symptoms at baseline assessment (approximately 4 months previously) predicted higher ITQ scores. After adjustment, there was no evidence that ITQ scores were associated with gender, key worker status or being at risk of severe infection due to a pre‐existing physical health condition.

## 4 DISCUSSION

### 4.1 Main findings

Although 40.10% (*n* = 630) of participants found some aspect of the COVID‐19 pandemic traumatic, only 5.28% (*n* = 83) reported a COVID‐19‐related traumatic exposure that was PTSD qualifying according to ICD‐11. The prevalence of probable ICD‐11 COVID‐19‐related PTSD was correspondingly low at 0.83% (*n* = 13). This estimate is considerably smaller than those reported previously. Some variability in estimates is expected across different countries and settings, and due to different diagnostic criteria and measurement tools. However, we believe our estimate was substantially lower since symptom measurement was anchored to a specific ICD‐11 PTSD qualifying event. The number of participants meeting symptom criteria for PTSD who reported a COVID‐19‐related traumatic experience that was not PTSD qualifying was 10.88% (*n* = 171). 15.7% of those who reported a PTSD qualifying trauma screened positive for probable COVID‐19 related PTSD.

Perhaps the most relevant comparisons with prevalence estimates in the general population are with two surveys that also used the ITQ (Karatzias et al., 2020; Shevlin et al., 2020). These ran in parallel yielding estimates of 16.79% in the United Kingdom (*N* = 2025) [10], and 17.7% in the Republic of Ireland (*N* = 1041) (Karatzias et al., 2020). The estimates were based on an instruction to complete the ITQ “in relation to your experience of the COVID‐19 pandemic.” The resulting prevalence estimates were thereby comparable to the estimate we reported based on PTSD symptom criteria alone (10.88%). According to this comparison and considering other studies that have reported much higher prevalence estimates (Yuan et al., 2021; Zhang et al., 2021), we did not find evidence that individuals with existing psychiatric disorders were more vulnerable to the development of COVID‐19‐related traumatic stress symptoms than the general population. The findings may even suggest that individuals with lived experience of psychiatric disorder are less vulnerable to the development of COVID‐19‐related PTSD. This could be due to a reduced likelihood of exposure to PTSD qualifying events, or significant prior experience of dealing with adversity and isolation.

The low prevalence of COVID‐19‐related PTSD within the sample was largely due to the very small number of PTSD qualifying events. A long‐standing debate surrounds the definition of the traumatic stressor criterion for PTSD (Alessi et al., 2013). Those in favor of a broad definition argue that too many restrictions may result in traumatic‐stress symptoms going untreated among people who would benefit from clinical intervention. Those wishing to retain restrictive criteria fear that broadening the definition risks over‐use of the diagnosis. PTSD never occurs spontaneously, and it is logical that this is reflected by diagnostic criteria. However, it has been argued that the gateway criterion should be based on data related to the frequency of PTSD symptoms occurring after different traumatic exposures. Although some work has been conducted in this area providing preliminary evidence of comparable levels of symptoms after PTSD qualifying versus nonqualifying events, results have been inconsistent, and it may not have received the research attention it deserves (Hyland et al., 2020). Uncertainty remains in terms of the clinical significance of traumatic stress symptoms that arise in the absence of a PTSD qualifying event, especially in the context of COVID‐19 (Norrholm et al., 2021). This is illustrated by the differential rates of PTSD qualifying events according to ICD‐11 and DSM‐5 criteria in our study and in previous studies. Nonetheless, valid prevalence estimates of COVID‐19‐related PTSD rely on adherence to current diagnostic criteria, and this has been neglected by studies to date.

In concordance with previous research on COVID‐19 and other outbreaks of infectious disease, we found evidence that traumatic stress symptoms were associated with younger age (Karatzias et al., 2020) lower‐income, and financial worries. Consistent with most previous studies, we found evidence of an association between lower levels of perceived social support and traumatic stress symptoms (Boyraz & Legros, 2020) but this contrasts with a study of psychiatric patients in China during the pandemic, which failed to find an association (Tang et al., 2021). Previous studies that investigated associations between COVID‐19‐related traumatic stress symptoms and gender, physical health and risk of severe infection, and key worker status reported mixed findings and we found no evidence of such associations in our study. Higher levels of anxiety and depression symptoms in the first survey predicted higher traumatic stress symptoms in the follow‐up survey approximately 5 months later. Caution is needed when interpreting these results since most participants did not meet diagnostic criteria for PTSD. These factors should be interpreted as predictive of *traumatic stress symptoms* rather than a diagnosis of PTSD.

### 4.2 Strengths and limitations

This is one of the few studies to explore COVID‐19‐related PTSD among people with existing psychiatric disorder. Unlike most studies, we determined whether COVID‐19‐related traumas were PTSD qualifying and anchored the measurement of symptoms to specific events. Experienced researchers independently grouped the traumas and judged whether they were PTSD qualifying. Unlike most previous studies, participants were only considered to fulfil the criteria for probable COVID‐19 related ICD‐11 PTSD if a qualifying trauma was reported, and criteria related to symptoms and functional impairment were met. This resulted in a lower but arguably more valid estimate than those derived with use of a DSM‐5 measurement tool such as the PTSD Checklist for DSM‐5 (PCL‐5; Blevins et al., 2015), that do not assess functional impairment.

The findings should be considered in the context of study limitations. First, we did not administer a diagnostic interview. However, the ITQ has good psychometric properties and is the gold standard self‐report measure of ICD‐11 PTSD (Cloitre et al., 2018). Second, we looked at factors associated with continuous ITQ scores, and it may be that different factors are associated with meeting symptom criteria for probable PTSD. Thirdly, due to the pandemic, the survey was online, which may have suboptimally represented older participants and participants from very low‐income households, who are less likely to be digitally active (Serafino, 2019). Additionally, only people who completed the baseline survey were invited to complete the trauma follow‐up. The impact of multiple traumas was not explored, and the extent of symptoms may have been under‐reported. This said, the measurement of PTSD with the ITQ should always be anchored to a single event. Further, it should be noted that screening negative on the ITQ anchored to specific traumatic events does not rule out the possibility that participants met criteria for PTSD to other COVID‐19‐related events. However, a high number of false negatives is unlikely, given that participants would have been most likely to meet criteria for PTSD in relation to the experience perceived as most troubling. It is also possible that we under‐estimated the number of PTSD qualifying events since some free‐text descriptions were too short or vague to be able to classify them as PTSD qualifying. Finally, the wide‐ranging recruitment strategies used to build the NCMH cohort, in addition to response biases associated with the follow‐up survey, resulted in a sample that is unlikely to be fully generalisable. Although it is a strength that the sample included participants with diagnoses such as personality disorder and schizophrenia, who are traditionally underrepresented in research (Martin et al., 2016), the findings should not be over‐interpreted. There are inherent difficulties in conducting research with a population with lived experience of psychiatric disorder who were exposed to a major public health crisis, which inevitably led to methodological compromises. This said, the findings are not invalidated by their limitations. It is also worth noting the sample was 95.41% White, which also limits generalisability. However, a high proportion of participants were recruited in Wales, which has a low level of ethnic diversity and an estimated 92.2% of the population identify as White Welsh/British (Office for National Statistics, 2019).

### 4.3 Clinical implications

Although few of the traumatic exposures were PTSD qualifying, 40% of participants reported events they perceived as traumatic. A knowledge of troubling aspects of the pandemic and responses to these is valuable and should inform response strategies to future pandemics. Many of the social distancing measures enacted to protect the physical health of the population caused psychological distress (Rodríguez‐Fernández et al., 2021). There is a need to strike the right balance between mitigating the spread of disease and preventing a parallel mental health pandemic. Our work did not find evidence of widespread COVID‐19‐related PTSD. However, it is important to note that in the context of such a widespread event, even a very small percentage of people with PTSD amounts to a significant mental health burden (Horesh & Brown, 2020). Given the often‐chronic nature of PTSD, identifying risk factors for its development is vital, whilst being cautious not to unnecessarily pathologise transient reactions. Clinical services would benefit from being trauma‐informed and equipped to deal with PTSD on its own or in combination with other conditions.

### 4.4 Research implications

There is a need to replicate this study in additional samples, both with and without lived experience of psychiatric disorder. In addition, future follow‐up of the cohort is required to establish whether effects dissipate, endure, or intensify over time. This may include participant follow‐up that utilises routinely collected health‐service data to minimise the bias associated with self‐report data. Studies using diagnostic interviews and robust methods of ascertaining exposure to COVID‐19‐related trauma would be desirable. Prevalence estimates of COVID‐19‐related PTSD in the general population have ranged from 1% to over 60% within study samples. Valid assessment of PTSD relies on the measurement of traumatic stress symptoms and functional impairment spanning the requisite timeframe for the estimate being derived. In the absence of research that utilises more robust methods, it is impossible to compare prevalence estimates of COVID‐19‐related PTSD across different countries, settings, or groups of impacted individuals. Without a change in direction, the true scale of PTSD in the wake of COVID‐19 will remain unknown and the over‐inflated prevalence estimates reported to date may preclude the identification and treatment of true cases. This may represent a particular issue among vulnerable populations such as those with lived experience of psychiatric disorders, who are at greater risk of PTSD going undetected and untreated (Zammit et al., 2018). It would also be valuable to determine the impact of the pandemic on those with pre‐exiting PTSD to other events since traumatic stress symptoms among these people may have been impacted. Given that the types of COVID‐19‐related traumas reported may be more likely to precipitate or exacerbate symptoms of anxiety and depression, these may be more worthy of attention, and the current focus on PTSD may detract from this. This said, we did not anchor measurement of current anxiety and depressive symptoms specifically to the pandemic, which is another consideration for future research.

## CONFLICTS OF INTEREST

The authors declare no conflicts of interest.

## Supporting information

Supporting information.

## Data Availability

The data that support the findings of this study is available from the National Centre for Mental Health (NCMH) who welcome proposals for collaboration.
