# Supplementary material for: COVID‐19‐related posttraumatic stress disorder in adults with lived experience of psychiatric disorder
Source: Depress Anxiety. 2022 May 10;39(7):564–72. doi: 10.1002/da.23262 (PMC9348441; doi:10.1002/da.23262)
Supplement: Supplementary file 1 — Supporting information. [file DA-39-564-s001.docx]

**Supplementary material**

**Mental health diagnoses that could be endorsed by participants in the baseline survey in response to the following questions:**

**Have you ever been diagnosed with or received treatment for a mental health condition?** [Yes, No, Not sure, Prefer not to say]

IF YES:

**What mental health conditions have you been diagnosed with or received treatment for?** (SELECT ALL THAT APPLY)

*Mood Disorders*

Depressive disorder (Depression, Major Depression)

Bipolar disorder (Manic depression)

Mania/Hypomania

Premenstrual dysphoric disorder (PMDD) / Premenstrual syndrome (PMS)

*Psychotic Disorders*

Schizophrenia

Schizoaffective disorder

Psychosis

*Anxiety Disorders*

Anxiety (Generalised Anxiety Disorder, GAD)

Agoraphobia

Panic disorder

Phobias

Obsessive Compulsive Disorder (OCD)

*Autistic Spectrum Disorders*

Autism

Asperger’s syndrome

*Eating Disorders*

Anorexia

Bulimia

Binge Eating Disorder

*Attention Deficit Hyperactivity Disorder (ADHD)*

*Post-Traumatic Stress Disorder (PTSD)*

*Complex Post-Traumatic Stress disorder (CPTSD)*

*Personality Disorders*

Borderline personality disorder (emotionally unstable personality disorder)

Other Personality Disorder

*Alcohol and drug problems*

Alcohol dependence / misuse

Dependence / misuse of other drugs

*Dementia*

*Perinatal/Postpartum disorders*

Mood disorder in pregnancy

Postpartum psychosis (Postnatal psychosis / Puerperal Psychosis)

Postnatal depression (Postpartum Depression)

**Risk factors that could be endorsed to indicate high risk of severe infection in the follow-up survey**

Based on what we know about Coronavirus (COVID-19) so far, doctors have advised that the group of people identified in the list below are at an increased risk of severe illness from Coronavirus (COVID-19). Do you have any of the following conditions?

- Have been a recipient of a solid organ transplant
- Are currently receiving treatment for a cancer diagnosis
- A serious respiratory condition (e.g cystic fibrosis, asthma or chronic obstructive pulmonary disease COPD)
- Have an increased risk of infection
- Heart disease
- Kidney disease
- Liver disease
- Chronic neurological condition (such as Parkinson’s disease, motor neurone disease or multiple sclerosis)
- Diabetes
- A weakened immune system due to a medical condition or medications (such as steroid tablets)
- Are currently pregnant
- Other- please specify:

**Predictors of non-response to COVID-19 trauma survey**

*Results of regression analyses (univariate)*

| Variable | OR | | 95% CI | | P |
| --- | --- | --- | --- | --- | --- |
| Age | 0.98 |  | | 0.90-0.99 | 0.000 |
| Gender | 0.83 |  | | 0.73-0.94 | 0.000 |
| Ever employed | 0.71 |  | | 0.62-0.80 | 0.000 |
| Minority ethnicity | 1.57 |  | | 1.18-2.09 | 0.002 |
| Diagnosis of bipolar disorder | 1.24 |  | | 1.06-1.46 | 0.008 |
| Diagnosis of schizophrenia | 1.36 |  | | 1.09-1.69 | 0.006 |
| Diagnosis of PTSD/CPTSD | 0.90 |  | | 0.77-1.04 | 0.173 |

Age – continuous; gender coded as 0 = male, 1 = female; ever employed coded as 1 = yes, 0 = no; minority ethnicity coded as 1 = yes, 0 = no; diagnosis of bipolar disorder coded as 1 = yes, 0 = no; diagnosis of schizophrenia coded as 1 = yes, 0 = no; diagnosis of PTSD/CPTSD coded as 1 = yes, 0 = no.
